# 3-Methyl-1*H*-pyrrolo[2,1-*c*][1,4]oxazin-1-one

**DOI:** 10.1107/S1600536810006951

**Published:** 2010-02-27

**Authors:** Salman Tariq Khan, Peng Yu, Erbin Hua, Syed Nawazish Ali, Mehrun Nisa

**Affiliations:** a Department of Pharmaceutical Engineering, Biotechnology College, Tianjin University of Science & Technology (TUST), Tianjin 300457, People’s Republic of China; b Department of Chemical and Biomolecular Engineering, Yonsei University, 262 Seongsanno, Seodaemun-gu, Seoul 120-749, Republic of Korea

## Abstract

In the title mol­ecule, C_8_H_7_NO_2_, all the non-H atoms lie essentially in the same plane (r.m.s. deviation = 0.019 Å) In the crystal structure, weak inter­molecular C—H⋯O inter­actions link mol­ecules into chains along [100]. In addition, there are π–π stacking inter­actions between mol­ecules related by the *c*-glide plane, with alternating centroid–centroid distances of 3.434 (2) and 3.639 (2) Å.

## Related literature

For the synthesis and applications of the title compound, see: Dumas *et al.* (1988 ▶); Micheli *et al.* (2008 ▶). For standard bond-length data, see: Allen *et al.* (1987 ▶).
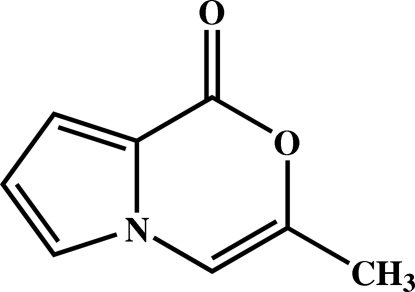


## Experimental

### 

#### Crystal data

C_8_H_7_NO_2_
*M* *_r_* = 149.15
Monoclinic, 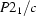

*a* = 6.915 (4) Å
*b* = 15.502 (8) Å
*c* = 7.024 (4) Å
β = 112.866 (8)°
*V* = 693.8 (6) Å^3^
*Z* = 4
Mo *K*α radiation
μ = 0.10 mm^−1^
*T* = 113 K
0.32 × 0.28 × 0.08 mm

#### Data collection

Rigaku Saturn CCD area-detector diffractometer
Absorption correction: multi-scan (*CrystalClear*; Rigaku, 2005 ▶) *T*_min_ = 0.967, *T*_max_ = 0.992
4630 measured reflections
1223 independent reflections
957 reflections with *I* > 2σ(*I*)
*R*_int_ = 0.044

#### Refinement

*R*[*F* ^2^ > 2σ(*F* ^2^)] = 0.035
*wR*(*F* ^2^) = 0.091
*S* = 1.01
1223 reflections
102 parameters
H-atom parameters constrained
Δρ_max_ = 0.23 e Å^−3^
Δρ_min_ = −0.19 e Å^−3^

### 

Data collection: *CrystalClear* (Rigaku, 2005 ▶); cell refinement: *CrystalClear*; data reduction: *CrystalClear*; program(s) used to solve structure: *SHELXS97* (Sheldrick, 2008 ▶); program(s) used to refine structure: *SHELXL97* (Sheldrick, 2008 ▶); molecular graphics: *PLATON* (Spek, 2009 ▶); software used to prepare material for publication: *CrystalClear*.

## Supplementary Material

Crystal structure: contains datablocks I, global. DOI: 10.1107/S1600536810006951/pv2260sup1.cif

Structure factors: contains datablocks I. DOI: 10.1107/S1600536810006951/pv2260Isup2.hkl

Additional supplementary materials:  crystallographic information; 3D view; checkCIF report

## Figures and Tables

**Table 1:** Hydrogen-bond geometry (Å, °)

*D*—H⋯*A*	*D*—H	H⋯*A*	*D*⋯*A*	*D*—H⋯*A*
C7—H7⋯O2^i^	0.95	2.52	3.252 (3)	134

Symmetry code: (i) 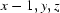.

**Fig. 1.**
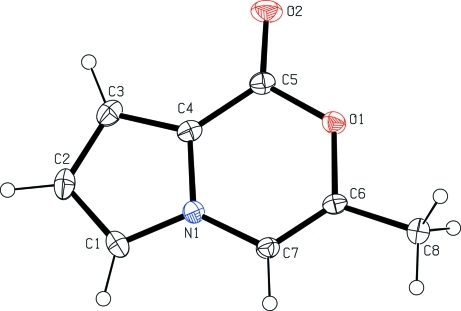
The molecular structure of the title compound with displacement ellipsoids drawn at the 30% probability level.

**Fig. 2.**
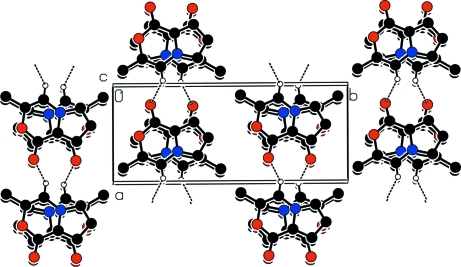
Part of the crystal structure of the title compound with weak C—H···O hydrogen bonds drawn as dashed lines.

## supplementary crystallographic information

### Comment

The preparation of the title compound was originally reported by Dumas (1988) as an intermediate in the synthesis of peramine. Recently, Micheli *et al.* (2008) used various analogues of this compound to synthesize a new series of pyrrolo[1,2-a]pyrazine compounds that are potent and selective non-competitive mGluR5 antagonists.

The crystal structure of the title compound is shown in Fig. 1. The bond lengths are as expected (Allen *et al.*, 1987). All the non-hydrogen atoms are essentially in the same plane (r.m.s. deviation = 0.019 Å). In the crystal structure, weak intermolecular C—H···O interactions link molecules into chains along [100] (Fig. 2). In addition, there are π–π stacking interactions with Cg1···Cg2(x,3/2-y,-1/2+z) = 3.434 (2) and Cg1···Cg2(x,3/2-y,1/2+z) = 3.639 (2) Å, where Cg1 and Cg2 are the centroids defined by rings atoms N1/C1—C4 and O1/C5/C4/N1/C7/C6, respectively.

### Experimental

A solution of 1-chloropropan-2-one (7.56 mL, 90 mmol) in acetone (50 ml) was dropwise added through a dropping funnel to a slurry of 2,2,2-trichloro-1-(lH-pyrrol-2-yl)ethanone (12.72 g, 60 mmol), potassium carbonate (24.84 g, 180 mmol) and acetone (150 ml) at room temperature in a 250 ml round-bottom flask. The reaction mixture was stirred at room temperature. After 24 h, the solid was removed by filtration and washed with acetone. The filtrate was concentrated under reduced pressure by rotary evaporator, the residue was partitioned between water and ethyl acetate (200 ml each) in a separatory funnel (500 ml). The organic layer was separated and the aqueous phase was washed with ethyl acetate (100 ml x 2). The combined organic layers were washed successively with water (100 ml x 3) and brine solution and dried over anhydrous MgSO_4_. After filtration, the solvent was removed by rotary evaporator to obtain the oily brown solid residue (13.0 g) which was purified by flash column chromatography (Petroleum ether: Ethyl acetate; 2:1) to afford the desired compound as pale yellow solid (5.1 g, 57%). The product was recrystallized in a mixture of petroleum ether and ethyl acetate (5:1). The colorless needles of the title compound were obtained by slow evaporation of solvent at room temperature. Melting point and NMR spectral data were consistent with the reported values (Dumas, 1988).

### Refinement

H atoms were placed in calculated positions with C—H = 0.95Å or C—H = 0.98Å for methyl H atoms and were included in the refinement in a riding-model approximation with U_iso_(H) = 1.2U_eq_(C) or 1.5U_eq_(C_methyl_).

### Crystal data

**Table:**

C_8_H_7_NO_2_	*F*(000) = 312
*M**_r_* = 149.15	*D*_x_ = 1.428 Mg m^−^^3^
Monoclinic, *P*2_1_/*c*	Mo *K*α radiation, λ = 0.71073 Å
Hall symbol: -P 2ybc	Cell parameters from 2364 reflections
*a* = 6.915 (4) Å	θ = 3.1–27.9°
*b* = 15.502 (8) Å	µ = 0.10 mm^−^^1^
*c* = 7.024 (4) Å	*T* = 113 K
β = 112.866 (8)°	Prism, colorless
*V* = 693.8 (6) Å^3^	0.32 × 0.28 × 0.08 mm
*Z* = 4	

### Data collection

**Table:**

Rigaku Saturn CCD area-detector diffractometer	1223 independent reflections
Radiation source: rotating anode	957 reflections with *I* > 2σ(*I*)
multilayer	*R*_int_ = 0.044
Detector resolution: 14.63 pixels mm^-1^	θ_max_ = 25.0°, θ_min_ = 3.4°
ω and φ scans	*h* = −8→8
Absorption correction: multi-scan (*CrystalClear*; Rigaku, 2005)	*k* = −12→18
*T*_min_ = 0.967, *T*_max_ = 0.992	*l* = −8→8
4630 measured reflections	

### Refinement

**Table:**

Refinement on *F*^2^	Secondary atom site location: difference Fourier map
Least-squares matrix: full	Hydrogen site location: inferred from neighbouring sites
*R*[*F*^2^ > 2σ(*F*^2^)] = 0.035	H-atom parameters constrained
*wR*(*F*^2^) = 0.091	*w* = 1/[σ^2^(*F*_o_^2^) + (0.0505*P*)^2^] where *P* = (*F*_o_^2^ + 2*F*_c_^2^)/3
*S* = 1.01	(Δ/σ)_max_ = 0.002
1223 reflections	Δρ_max_ = 0.23 e Å^−^^3^
102 parameters	Δρ_min_ = −0.19 e Å^−^^3^
0 restraints	Extinction correction: *SHELXL97* (Sheldrick, 2008), Fc^*^=kFc[1+0.001xFc^2^λ^3^/sin(2θ)]^-1/4^
Primary atom site location: structure-invariant direct methods	Extinction coefficient: 0.025 (6)

### Special details

**Table:**

Geometry. All esds (except the esd in the dihedral angle between two l.s. planes) are estimated using the full covariance matrix. The cell esds are taken into account individually in the estimation of esds in distances, angles and torsion angles; correlations between esds in cell parameters are only used when they are defined by crystal symmetry. An approximate (isotropic) treatment of cell esds is used for estimating esds involving l.s. planes.
Refinement. Refinement of *F*^2^ against ALL reflections. The weighted *R*-factor wR and goodness of fit *S* are based on *F*^2^, conventional *R*-factors *R* are based on *F*, with *F* set to zero for negative *F*^2^. The threshold expression of *F*^2^ > σ(*F*^2^) is used only for calculating *R*-factors(gt) etc. and is not relevant to the choice of reflections for refinement. *R*-factors based on *F*^2^ are statistically about twice as large as those based on *F*, and *R*- factors based on ALL data will be even larger.

### Fractional atomic coordinates and isotropic or equivalent isotropic displacement parameters (Å^2^)

**Table:**

	*x*	*y*	*z*	*U*_iso_*/*U*_eq_	
O1	0.45807 (13)	0.61029 (5)	0.34499 (13)	0.0259 (3)	
O2	0.78058 (13)	0.65952 (7)	0.42312 (15)	0.0384 (3)	
N1	0.29846 (17)	0.77385 (7)	0.30630 (16)	0.0221 (3)	
C1	0.2544 (2)	0.85965 (8)	0.2913 (2)	0.0287 (4)	
H1	0.1204	0.8847	0.2615	0.034*	
C2	0.4378 (2)	0.90383 (9)	0.32687 (19)	0.0323 (4)	
H2	0.4525	0.9647	0.3252	0.039*	
C3	0.5987 (2)	0.84336 (9)	0.3659 (2)	0.0304 (4)	
H3	0.7423	0.8555	0.3960	0.037*	
C4	0.5104 (2)	0.76289 (8)	0.35257 (18)	0.0237 (4)	
C5	0.5972 (2)	0.67754 (8)	0.3770 (2)	0.0250 (4)	
C6	0.24605 (19)	0.62433 (8)	0.30253 (19)	0.0228 (3)	
C7	0.1665 (2)	0.70294 (8)	0.28336 (19)	0.0235 (3)	
H7	0.0216	0.7111	0.2544	0.028*	
C8	0.1316 (2)	0.54197 (8)	0.2877 (2)	0.0316 (4)	
H8A	−0.0147	0.5542	0.2654	0.047*	
H8B	0.1981	0.5093	0.4162	0.047*	
H8C	0.1357	0.5080	0.1716	0.047*	

### Atomic displacement parameters (Å^2^)

**Table:**

	*U*^11^	*U*^22^	*U*^33^	*U*^12^	*U*^13^	*U*^23^
O1	0.0236 (5)	0.0252 (5)	0.0306 (5)	0.0023 (4)	0.0125 (4)	0.0013 (4)
O2	0.0227 (6)	0.0449 (7)	0.0502 (7)	0.0045 (4)	0.0170 (5)	0.0071 (5)
N1	0.0238 (6)	0.0216 (6)	0.0213 (6)	0.0004 (4)	0.0093 (4)	0.0011 (4)
C1	0.0359 (8)	0.0227 (7)	0.0276 (7)	0.0059 (6)	0.0123 (6)	0.0015 (6)
C2	0.0449 (10)	0.0223 (7)	0.0268 (8)	−0.0076 (7)	0.0110 (7)	−0.0004 (6)
C3	0.0296 (8)	0.0338 (8)	0.0265 (7)	−0.0083 (6)	0.0093 (6)	0.0009 (6)
C4	0.0212 (7)	0.0299 (8)	0.0197 (7)	−0.0018 (6)	0.0076 (5)	0.0018 (6)
C5	0.0225 (8)	0.0307 (8)	0.0235 (7)	−0.0006 (6)	0.0108 (6)	0.0024 (6)
C6	0.0187 (7)	0.0289 (8)	0.0212 (7)	−0.0003 (6)	0.0081 (5)	−0.0003 (6)
C7	0.0197 (7)	0.0258 (7)	0.0254 (7)	−0.0008 (6)	0.0091 (5)	0.0006 (6)
C8	0.0307 (8)	0.0250 (7)	0.0375 (8)	−0.0026 (6)	0.0114 (7)	−0.0009 (6)

### Geometric parameters (Å, °)

**Table:**

O1—C5	1.3767 (16)	C3—C4	1.3761 (19)
O1—C6	1.3950 (17)	C3—H3	0.9500
O2—C5	1.2128 (16)	C4—C5	1.4356 (19)
N1—C1	1.3596 (18)	C6—C7	1.3223 (19)
N1—C4	1.3826 (19)	C6—C8	1.4844 (18)
N1—C7	1.3976 (18)	C7—H7	0.9500
C1—C2	1.376 (2)	C8—H8A	0.9800
C1—H1	0.9500	C8—H8B	0.9800
C2—C3	1.398 (2)	C8—H8C	0.9800
C2—H2	0.9500		
			
C5—O1—C6	121.78 (10)	O2—C5—O1	117.45 (12)
C1—N1—C4	108.89 (11)	O2—C5—C4	126.13 (12)
C1—N1—C7	130.07 (12)	O1—C5—C4	116.42 (12)
C4—N1—C7	121.03 (11)	C7—C6—O1	121.79 (12)
N1—C1—C2	108.03 (13)	C7—C6—C8	126.58 (13)
N1—C1—H1	126.0	O1—C6—C8	111.61 (11)
C2—C1—H1	126.0	C6—C7—N1	119.06 (13)
C1—C2—C3	108.01 (13)	C6—C7—H7	120.5
C1—C2—H2	126.0	N1—C7—H7	120.5
C3—C2—H2	126.0	C6—C8—H8A	109.5
C4—C3—C2	107.21 (13)	C6—C8—H8B	109.5
C4—C3—H3	126.4	H8A—C8—H8B	109.5
C2—C3—H3	126.4	C6—C8—H8C	109.5
C3—C4—N1	107.85 (12)	H8A—C8—H8C	109.5
C3—C4—C5	132.32 (14)	H8B—C8—H8C	109.5
N1—C4—C5	119.83 (11)		
			
C4—N1—C1—C2	0.31 (14)	C6—O1—C5—C4	−3.46 (18)
C7—N1—C1—C2	179.99 (12)	C3—C4—C5—O2	2.3 (3)
N1—C1—C2—C3	−0.36 (16)	N1—C4—C5—O2	−177.76 (12)
C1—C2—C3—C4	0.26 (16)	C3—C4—C5—O1	−177.79 (13)
C2—C3—C4—N1	−0.07 (15)	N1—C4—C5—O1	2.12 (18)
C2—C3—C4—C5	179.84 (13)	C5—O1—C6—C7	2.52 (18)
C1—N1—C4—C3	−0.15 (14)	C5—O1—C6—C8	−176.31 (11)
C7—N1—C4—C3	−179.86 (11)	O1—C6—C7—N1	0.00 (19)
C1—N1—C4—C5	179.92 (12)	C8—C6—C7—N1	178.64 (12)
C7—N1—C4—C5	0.22 (18)	C1—N1—C7—C6	179.04 (12)
C6—O1—C5—O2	176.42 (11)	C4—N1—C7—C6	−1.32 (18)

### Hydrogen-bond geometry (Å, °)

**Table:**

*D*—H···*A*	*D*—H	H···*A*	*D*···*A*	*D*—H···*A*
C7—H7···O2^i^	0.95	2.52	3.252 (3)	134

Symmetry codes: (i) *x*−1, *y*, *z*.

## References

[bb1] Allen, F. H., Kennard, O., Watson, D. G., Brammer, L., Orpen, A. G. & Taylor, R. (1987). *J. Chem. Soc. Perkin Trans. 2* pp. S1–19.

[bb2] Dumas, D. J. (1988). *J. Org. Chem.* **53**, 4650–4653.

[bb3] Micheli, F., Bertani, B., Bozzoli, A., Crippa, L., Cavanni, P., Di Fabio, R., Donati, D., Marzorati, P., Merlo, G., Paio, A., Perugini, L. & Zarantonello, P. (2008). *Bioorg. Med. Chem. Lett.* **18**, 1804–1809. 10.1016/j.bmcl.2008.02.024 18304814

[bb4] Rigaku (2005). *CrystalClear.* Rigaku Corporation, Tokyo, Japan.

[bb5] Sheldrick, G. M. (2008). *Acta Cryst.* A**64**, 112–122. 10.1107/S0108767307043930 18156677

[bb6] Spek, A. L. (2009). *Acta Cryst.* D**65**, 148–155. 10.1107/S090744490804362X PMC2631630 19171970

